# Mild cold stress specifically disturbs clustering movement of DFCs and sequential organ left-right patterning in zebrafish

**DOI:** 10.3389/fcell.2022.952844

**Published:** 2022-09-23

**Authors:** Min Liu, Xinyu Zou, Mao Fu, Xinping Bai, Yongyan Zhao, Xin Chen, Xiaoyu Wang, Peijian Wang, Sizhou Huang

**Affiliations:** ^1^ Development and Regeneration Key Laboratory of Sichuan Province, Department of Anatomy and Histology and Embryology, School of Basic Medical Sciences, Chengdu Medical College, Chengdu, China; ^2^ Department of Cardiology, the First Affiliated Hospital, Chengdu Medical College, Chengdu, China; ^3^ School of Pharmacy, Chengdu Medical College, Chengdu, China; ^4^ School of Clinical Medicine, Chengdu Medical College, Chengdu, China; ^5^ School of Public Health, Chengdu Medical College, Chengdu, China; ^6^ School of Biomedical Sciences, Chengdu Medical College, Chengdu, China

**Keywords:** cold stress, DFCs, LR patterning defect, zebrafish, Cdh1, Cdh2

## Abstract

In poikilothermic animals, the distinct acclimatization ability of different organs has been previously addressed, while the tissue-specific role of cold stress in early development is largely unknown. In this study, we discovered that despite its role in delaying embryonic development, mild cold stress (22°C) does not disturb multiple-organ progenitor specification, but does give rise to organ left-right (LR) patterning defects. Regarding the mechanism, the data showed that mild cold stress downregulated the expression of cell-adhesion genes *cdh1* and *cdh2* during gastrulation, especially in dorsal forerunner cells (DFCs), which partially disturbed the clustering movement of DFCs, Kupffer’s vesicle (KV) morphogenesis, and ciliogenesis. As a result, the defects of KV/cilia disrupted asymmetric *nodal* signaling and subsequent heart and liver LR patterning. In conclusion, our data novelly identified that, in early development, DFCs are more sensitive to mild cold stress, and mild cold stress repressed the expression of cell adhesion-related gene *cdh1* and *cdh2*. This role partially disturbed the clustering movement of DFCs, which resulted in defective KV/cilia development and sequential organ LR patterning defects.

## Introduction

Endotherms and heterothermic vertebrates use distinct methods to adapt to cold ambient temperatures (Lu et al., 2019). To maintain a consistent body temperature throughout a range of external environmental temperature changes, endotherms have evolved many conscious behaviors and non-conscious physiological responses such as huddling, increasing activity, constricting blood flow, shivering, and hibernation (Carey et al., 2003; Messmer et al., 2014; Lu et al., 2019). Heterothermic vertebrates such as fish have none of these methods to maintain a consistent temperature, but they have evolved some mechanisms related to cold adaptation/resistance such as the alteration of cell-membrane fluidity (Johnston and Roots, 1964), synthesis of molecular chaperones (Reid et al., 2022), and antifreeze proteins (Cao et al., 2016). By using these mechanisms, fish can live in a range of cold ambient temperatures (Fangue et al., 2014; Chen et al., 2019; Reid et al., 2022).

Despite vertebrates having adapted a series of mechanisms to maintain life, the function of internal organs and organ development are still affected to some degree (Serrat et al., 2008; Scott and Johnston, 2012; Hu et al., 2015; Zhang et al., 2018). In mammals, two critical examples are as follows: low temperature directly decreases cartilage growth and results in shorter limbs, and cold stress also disturbs neural development (Xia et al., 2018), even though cold-induced genes are employed to protect against abnormal neurodevelopment and neurodegeneration (Peretti et al., 2015). A negative effect of low temperature on organ development has also been observed in fish (Reid et al., 2022). In zebrafish, treatment with cold stress at the larval stage affects thermal sensitivity and acclimation of swimming performance in the adult stage, resulting from a variation in the fiber type composition in swimming muscles (Scott and Johnston, 2012). Furthermore, low ambient temperature (24°C) also reduces the survival ratio of zebrafish larvae and disturbs innate immune processes upon exposure to lipopolysaccharide (LPS) (Zhang et al., 2018). To further understand the mechanisms by which fish cope with cold stress and how organ development/function was affected, several studies have been carried out (Long et al., 2012; Long et al., 2013; Hu et al., 2015; Hu et al., 2016). Interestingly, a study on the common carp discovered that a large number of genes exhibited highly tissue-specific expression changes upon cold stress (Gracey et al., 2004) and this result was also observed in zebrafish (Hu et al., 2015). These studies imply that different tissues/cells display distinct responses to cold stress.

In previous literature, although the influences of cold stress on organ function (Uliano et al., 2010; Lin et al., 2014; Liu C. et al., 2019) and development (Bacchetta et al., 2020) were addressed, no studies have yet clarified whether cold stress influences cellular activity during early development. The zebrafish embryo is ectogenetic and its developmental temperature is easily manipulated (Abozaid et al., 2020; Urushibata et al., 2021); therefore, we chose the zebrafish to analyze the role of cold stress on early embryonic development. In the current study, we found that when embryos were treated with different cold stress from sphere to the 2-somite stage (SS), they were subsequently more sensitive to serious cold stress (16–19°C), displaying embryonic mortality or malformation. On the contrary, mild cold stress (22°C) did not disturb cell-fate determination or organ size but specifically led to defects in DFC clustering, KV morphogenesis, ciliogenesis, and sequential organ LR defect. The mechanism behind this downregulation of the expression of cell adhesion-related genes *cdh1* and *cdh2* mediated, at least partially, this process upon mild cold stress.

## Methods

### Zebrafish

Zebrafish wild type (AB), Tg (*flk1*:GFP) (Allen et al., 2017), Tg (*huc*:GFP) (Huang et al., 2019), Tg (*cmcl2*:GFP) (Zhu et al., 2019), Tg (*fabp10*:GFP) (Zhu et al., 2019), and Tg (*sox17*:GFP) (Liu J. et al., 2019) lines were maintained at 28.5°C. The embryonic stage was determined according to external morphology as previously described (Kimmel et al., 1995).

### Cold stress treatment

The embryos were collected at 0.5 h post-fertilization (hpf) and incubated at 28.5°C. At the sphere stage, the embryos were divided into six groups; one group was incubated at 28.5°C as the control and the other groups were incubated at 26°C, 24°C, 22°C, 19°C, and 16°C, respectively. When the embryos developed up to 2-SS, they were transferred to 28.5°C for further stage-specific incubation.

### Whole-mount *in situ* hybridization (WISH)

Embryos used for whole-mount *in situ* hybridization (WISH) were collected at the desired stages and fixed in 4% PFA at 4°C overnight. The embryos were then washed with PBST (twice, 5 min each), dehydrated with MeOH (100%), and stored in MeOH at −20°C. WISH followed the experimental procedure described previously (Huang et al., 2011). The antisense probes *vox*, *papc*, *her4*, *my17*, *fabp10*, *spaw*, *lefty1*, *lefty2*, *shh*, *sox17*, and *sox32* were prepared as previously described (Zhu et al., 2019). The *cdh1* and *cdh2* probes were synthesized using PCR products as templates. To prepare the templates, the CDs of *cdh1* and *cdh2* were amplified individually using commercial kits (Prim STAR Max Premix, Takara, No. R045A). After obtaining the PCR product for each gene, part of the PCR product was used for sequencing to confirm its suitability, and the remainder was used as templates to synthesize antisense probes. The primers were as follows: *cdh1*_F: 5′-CCA​CCT​GAG​TTT​ATT​CCA​AAG​GAG-3′, *cdh1*_R: 5′-GAT​TTA​GGT​GAC​ACT​ATA​GAA​TCT​TAA​TCC​TCT​CCT​CCT​CCA​TAC​ATG-3′. *Cdh2*_F: 5′-CAT​CAC​CGA​GGG​ACA​AGT​TCT​G-3′, *cdh2*_R:5′-GATTTAGGTGACACTATAGAATGGCACTTCATTGTCAGCGAC-3′.

### Immunostaining

Immunostaining was performed as previously reported (Zhu et al., 2019). Briefly, the embryos were fixed with 4% PFA at 4°C overnight, then dehydrated through a gradient of methanol, and stored at -20°C. After methanol/PBST gradient rehydration, PBTN (4% BSA, 0.02% NaN3, PT) was added and the embryos were incubated at 4°C for 3 h. Subsequently, the α-tubulin antibody (Sigma, T7451, diluted with PBTN at 1:100) was added and incubated overnight on a shaker at 4°C. The next day, the embryos were washed with PT (0.3% triton-X-100, in 1X PBS) four times (30 min each), and the second antibody goat anti-mouse IgG (H + L) Alexa Fluor 594 (Sigma, SAB4600105) was added (diluted with PBTN at 1:500) for overnight incubation (in darkness). Finally, the embryos were rinsed with PT >6 times (30 min each) and imaging was performed.

### Plasmid construction

Total RNA was extracted from zebrafish embryos at the bud stage according to the reagent instructions (TRIzol, Ambion No. 15596–026). cDNA was prepared using a Revert Aid First Strand cDNA Synthesis Kit (Fermentas No.K1622) according to the manufacturer’s instructions. The CDS of *cdh1* and *cdh2* were individually amplified using PCR (Prim STAR Max Premix Takara No. R045A) and cloned into the vector PCS^2+^ to generate the expression constructs (5x In-Fusion HD Enzemy Premix, Takara, No. 639649). The primers for cloning were as follows:

PCS^2+^_F: 5′-CTC​GAG​CCT​CTA​GAA​CTA​TAG​TG-3′,

PCS^2+^_R: 5′-TGG​TGT​TTT​CAA​AGC​AAC​GAT​ATC​G-3′,

*cdh1_*F: 5′-GCT​TTG​AAA​ACA​CCA​CTT​GTA​GCG​AGT​CAA​ATG​GCT​TG-3′,

*cdh1_*R: 5′-TTC​TAG​AGG​CTC​GAG​CTT​AAT​CCT​CTC​CTC​CTC​CAT​ACA​TG-3′,

*cdh2_*F: 5′-GCT​TTG​AAA​ACA​CCA​CGG​AAT​TTA​AAC​GAT​GTA​CCC​CTC​C-3′,

*cdh2*_R: 5′-TTC​TAG​AGG​CTC​GAG​GTG​ATG​GCG​TAA​GCT​AGT​CGT​C-3'.

### mRNA injection

The *cdh1* and *cdh2* mRNAs were synthesized *in vitro* using an mMESSAGE Kit (AM1340, Ambion). The concentration for mRNA injection was as follows: *cdh1* mRNA, 30 ng/μl; *cdh2* mRNA, 30 ng/μl. The *cdh1* and *cdh2* mRNA were mixed and injected into the yolk of zebrafish embryos at the 256–512 cell stage.

### Imaging

Images of the whole-mount *in situ* hybridization (in 100% glycerol) were taken with an OLYMPUS SZX16 at room temperature. The live embryos of transgenic line Tg (*sox17*:GFP) were placed in 1% low-point melting agarose (LPM agarose) and DFCs were photographed using a confocal microscope (OLYMPUS Fluview FV1000). For imaging of the immuno-stained embryos, they were adjusted to the correct orientation in 100% glycerol and then the cilia were photographed using a confocal microscope (OLYMPUS Fluview FV1000).

### Statistical analysis

The data were statistically analyzed using Graphpad Prism 9 and ImageJ software. The cilia length was measured using a confocal microscope (OLYMPUS Fluview FV1000). The statistical results were the mean ± SEM of three independent experiments.

### Ethics statement

The study was approved by the Institutional Review Board of Chengdu Medical College (SYXK(川)2015–196), date of approval: 22 July 2015), and zebrafish were maintained in accordance with the Guidelines of Experimental Animal Welfare from the Ministry of Science and Technology of People’s Republic of China (2006).

## Results

### Early embryos are more sensitive to cold stress

Adult zebrafish are reported to tolerate a wide range of temperatures from 6.7 to 41.7°C, with an optimal temperature of 28 C (Hu et al., 2015). Several previous studies used a narrow range of temperature (16–32 C) to address the influence of cold stress on organ development in the larval stage of zebrafish (Long et al., 2013; Hu et al., 2015), while no literature has addressed the role of cold stress on early embryonic development. To reveal whether cold stress influences more early developmental events, we narrowed the range of cold temperatures and selected 16°C, 19°C, and 22°C as ambient temperatures for embryonic development (Supplementary Figure S1A). Because most cell-fate determination occurs as early as the beginning of gastrulation (Pinheiro and Heisenberg, 2020), and during gastrulation the endoderm, mesoderm, and ectoderm are delaminated (Liu et al., 2009), cold stress treatment was applied from the sphere stage to 2-SS to analyze whether cold stress disrupted early developmental events (Supplementary Figure S1A). The data showed that the embryos displayed varying degrees of developmental delay under different types of cold stress (data not shown). In addition, when the embryos were treated at 16°C, 98% of them displayed embryonic malformation at the shield stage and eventually died at 2-SS (Supplementary Figure S1B, Supplementary Table S1). When the embryos were treated at 19°C, only 17% of embryos displayed embryonic malformation at the shield stage, and the ratio of mortality was only 36% at 2-SS (Supplementary Table S1). However, when the treatment temperature rose to 22°C, no obvious embryonic malformation or embryonic mortality was observed (Supplementary Table S1). Furthermore, there was no distinct phenotype in appearance at the shield stage, 24-SS, or protruding mouth stage when compared with controls (Supplementary Figure S2). The results indicated that early-stage embryos were more sensitive to cold stress, and mild cold stress (22°C) did not lead to embryonic malformation at this stage.

### Mild cold stress specifically results in organ LR patterning

**FIGURE 1:**
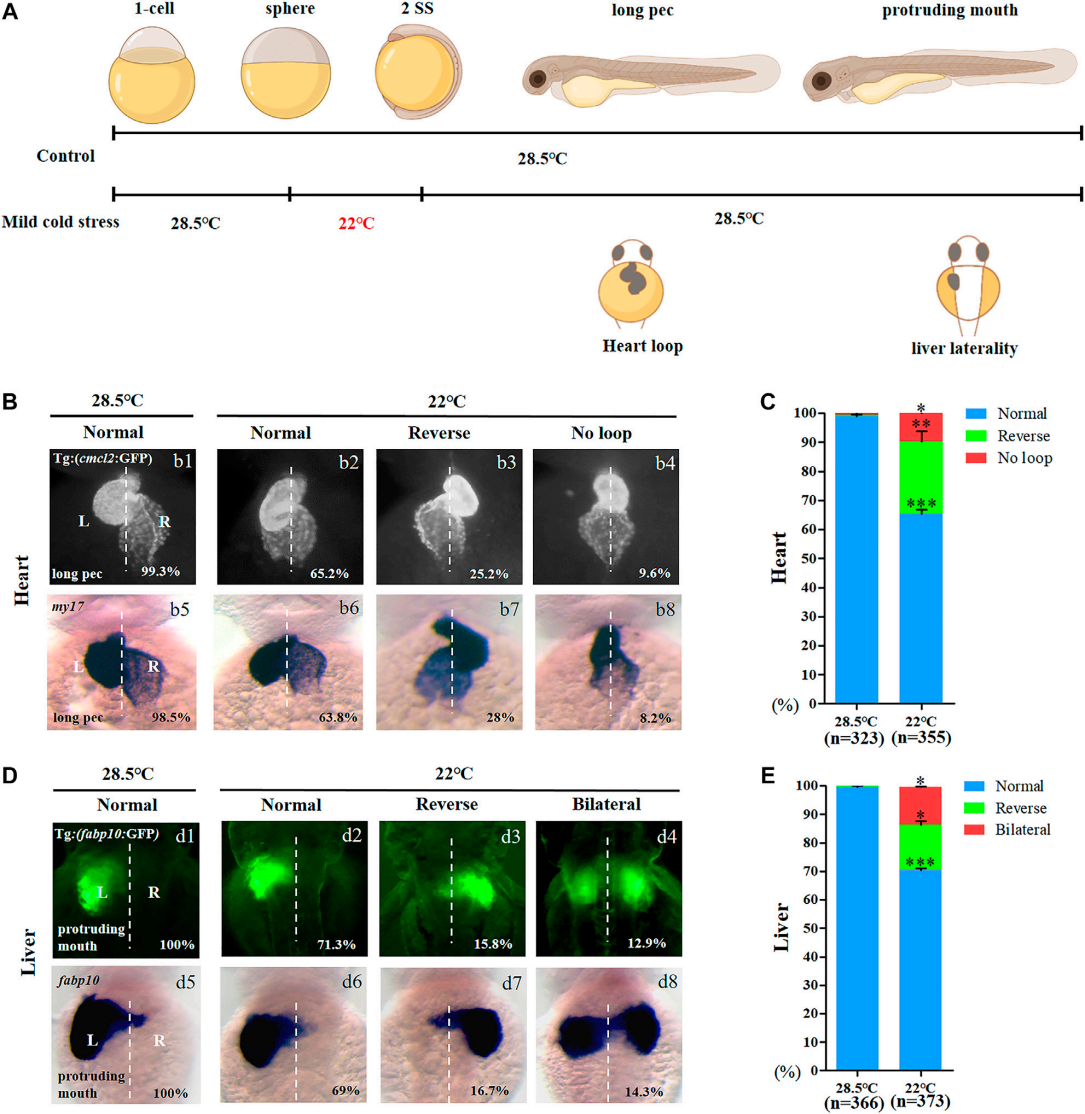
Organ left-right patterning defects in embryos treated with mild cold stress. **(A)** Stage and time diagram of treatment with mild cold stress. **(B)** Heart morphogenesis in Tg (cmcl2:GFP) transgenic line and wild-type embryos upon cold stress. b1, normal-loop at 28.5°C (99.3%, *n* = 160); b2, normal-loop at 22°C (65.2%, *n* = 180, *p* < 0.001); b3, reversed-loop at 22°C (25.2%, *n* = 180, *p* < 0.01); b4, no loop at 22°C (9.6%, *n* = 180, *p* < 0.04); b5, normal-loop at 28.5°C (98.5%, n = 163); b6, normal-loop at 22°C (63.8%, *n* = 175, *p* < 0.001); b7, reversed-loop at 22°C (28%, *n* = 175, *p* < 0.01); b8, no loop at 22°C (8.2%, *n* = 175, *p* < 0.04). **(C)** Percentages of normal looping, reversed looping, and no looping of the heart in embryos treated at 28.5 and 22°C. A statistically significant difference (*p* < 0.05) could be seen in embryos treated at 28.5 vs 22°C. **(D)** Mild cold stress was found to cause liver LR defects using Tg (fabp10:GFP) and fabp10 probe staining. d1, normal liver at 28.5°C (100%, *n* = 192); d2, normal liver at 22°C (71.3%, *n* = 201, *p* < 0.001); d3, reversed liver at 22°C (15.8%, n = 201, *p* < 0.03); d4, liver bifida at 22°C (12.9%, *n* = 201, *p* < 0.05); d5, normal liver at 28.5°C (100%, n = 174); d6, normal liver at 22°C (69%, *n* = 172, *p* < 0.001); d7, reversed liver at 22°C (16.7%, *n* = 172, *p* < 0.03); d8, liver bifida at 22°C (14.3%, *n* = 172, *p* < 0.05). **(E)** Percentages of normal liver, reversed liver, and liver bifida in embryos treated at 28.5 and 22°C. A statistically significant difference (*p* < 0.05) could be seen in embryos treated with 28.5 vs 22°C. “*”*p* < 0.05; “**“*p* < 0.03; and “***“*p* < 0.001.

Although mild cold stress treatment did not give rise to embryonic malformation, its role in organ development was not ruled out. To evaluate this possibility, the development of the heart, blood vessels, liver, and neurons was analyzed when mild cold stress was applied (Figure 1A). When compared with the control, the sizes of the liver and heart were normal (Figures 1B,D), and no defects were observed in the development of blood vessels and neurons (Supplementary Figure S3). In addition, the expression of early markers of the endoderm, mesoderm, and ectoderm was also unchanged at the early stage (Supplementary Figure S4). Interestingly, the heart of embryos treated with mild cold stress showed an LR patterning defect: 25.2 and 9.6% of embryos displayed reversed-loop heart and no-loop heart, respectively (Figure 1B, b1–b4, C). To confirm the phenotype of the heart LR patterning defect, the expression of *my17*, the heart-specific gene, was examined for wild-type embryos when mild cold stress was applied. The result was consistent with that in Tg (*cmcl2*:GFP) transgenic embryos (Figure 1B, b5–b8, C). Similarly, under mild cold stress, we observed that the ratio of embryos displaying right-sided liver and liver bifida was 15.8 and 12.9%, respectively; only 71.3% of embryos displayed normal liver location, which was far lower than that in controls (100%) (Figure 1D, d1–d4, E). The liver LR patterning defect was also confirmed in wild-type embryos (Figure 1D, d5–d8, E). The aforementioned results suggested that, at the gastrulation stage, mild cold stress specifically disturbed organ LR patterning.

### Mild cold stress disrupts *Nodal/spaw* signaling asymmetry

**FIGURE 2:**
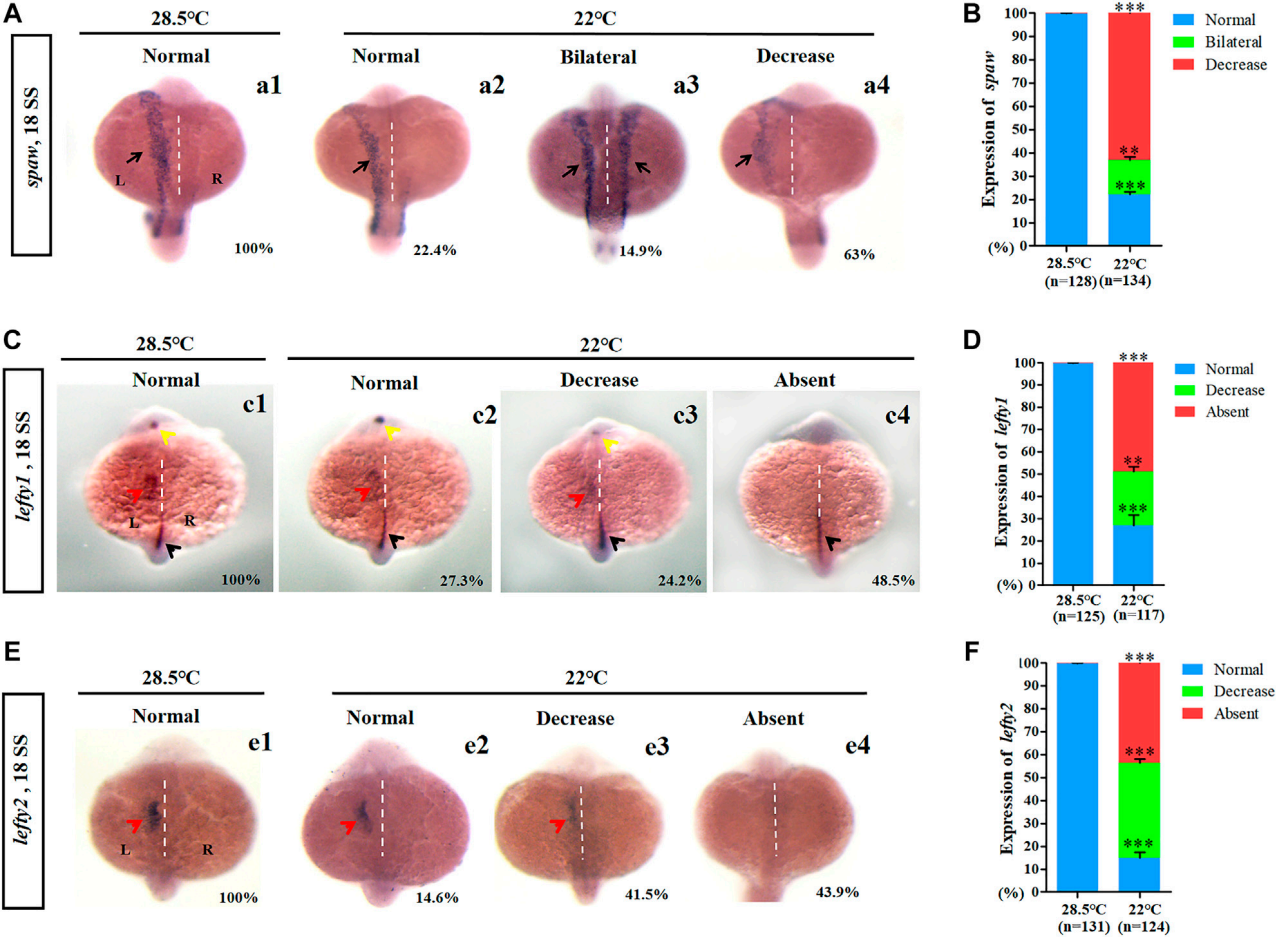
Expression of left-sided *Nodal* signaling in control and embryos treated with mild cold stress. **(A,B)** Expression of *spaw*. a1, left-side *spaw* at 28.5°C (100%, *n* = 128); a2, left-side *spaw* at 22°C (22.4%, *n* = 134, *p* < 0.001); a3, bilateral *spaw* at 22°C (14.9%, *n* = 134, *p* < 0.03); a4, decreased expression of *spaw* at 22°C (63%, *n* = 134, *p* < 0.001). **(C,D)** *Lefty1* is expressed in the left telencephalon (yellow arrow), left heart field (red arrow), trunk midline, and tail midline (black arrow). c1, normal *lefty1* at 28.5°C (100%, *n* = 125); c2, normal *lefty1* at 22°C (27.3%, *n* = 117, *p* < 0.001); c3, decreased expression of *lefty1* at 22°C (24.2%, *n* = 117, *p* < 0.01); c4, absent *lefty1* at 22°C (48.5%, *n* = 117, *p* < 0.002). **(E,F)** *Lefty2* is expressed in left heart field (red arrow) at 18 SS. e1, normal *lefty2* at 28.5°C (100%, *n* = 131); e2, normal *lefty2* at 22°C (14.6%, *n* = 124, *p* < 0.001); e3, decreased expression of *lefty2* at 22°C (41.5%, *n* = 124, *p* < 0.001); e4, absent *lefty2* at 22°C (43.9%, *n* = 124, *p* < 0.001). “**“*p* < 0.03; “***“*p* < 0.001.

In zebrafish, asymmetric expression of *Nodal/spaw* in the lateral plate mesoderm (LPM) is required for normal LR patterning (Levin, 2005; Matsui and Bessho, 2012). To study how mild cold stress leads to LR patterning defects in the heart and liver, the expression of *Nodal/spaw* was examined in embryos treated with mild cold stress. Data showed that the expression of *Nodal*/*spaw* was greatly changed upon mild cold stress: left-sided expression with normal level (22.4%), bilateral expression (14.9%), and left-side expression with decreased level (63%) (Figures 2A,B). To further confirm that the expression of *Nodal/spaw* was changed, the expression of *lefty1* and *lefty2*, two downstream genes of *Nodal/spaw* were examined. The expression of *lefty1* and *lefty2* was greatly downregulated upon mild cold stress (Figures 2C–F). In addition, in the diencephalon and heart field, the expression of *lefty1* was absent in 48.5% of embryos upon mild cold stress (Figure 2C c4), and the expression of *lefty2* was also absent in 43.9% of embryos (Figure 2E e4). Briefly, these results suggested that the expression of *Nodal/spaw* signaling was disturbed and this type of disruption may mediate organ LR defects in embryos treated with mild cold stress.

### Mild cold stress leads to defects of KV morphogenesis and ciliogenesis

**FIGURE 3:**
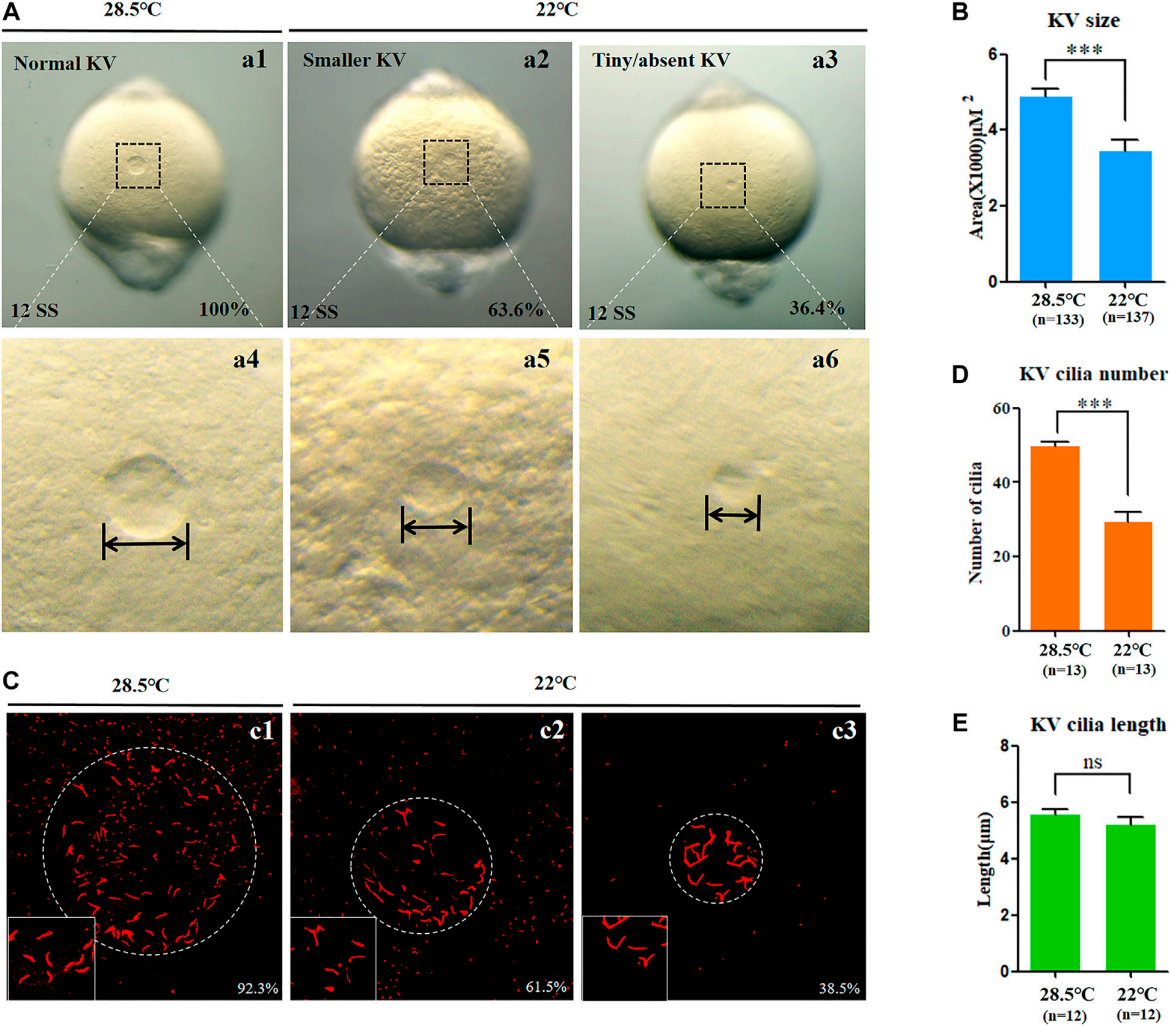
KV morphogenesis and ciliogenesis in embryos treated with mild cold stress. **(A,B)** Morphology of KV. a1, normal KV at 28.5°C (100%, *n* = 133); a2, smaller KV at 22°C (63.6%, *n* = 137, *p* < 0.001); a3, tiny/absent KV at 22°C (36.4%, *n* = 137, *p* < 0.002). **(C)** Number and length of cilia. C1, cilia at 28.5°C (92.3%, *n* = 13); c2, cilia at 22°C (61.5%, *n* = 13, *p* < 0.001); c3, cilia at 22°C (38.5%, *n* = 13, *p* < 0.003). **(D)** Statistical chart for cilia number in KV. *n* = 13, *p* < 0.001 **(E)** Statistical chart for cilia length in KV. *n* = 12. Ns, not significant; “***“*p* < 0.001.

In zebrafish, the function of cilia in Kupffer’s vesicles (KV) is critical for initiating the expression of left-sided *Nodal/spaw* in LPM (Essner et al., 2005; Kramer-Zucker et al., 2005). Furthermore, the embryonic midline plays an important role in preventing the transmission of left-sided signals from the left side to the right side of the embryos (Burdine and Grimes, 2016); any disruption to them can lead to organ LR patterning defects. Thus, we observed whether the development of midline, KV morphogenesis, and ciliogenesis was defected upon mild cold stress. The data showed that, under mild cold stress, the structure of the midline in living embryos was normal at 18 SS (Supplementary Figure S5A), and the expression of midline markers *lefty1* and *shh* in the midline was intact (Supplementary Figures S5B,C). These data suggested that the organ LR defects caused by mild cold stress were not due to midline defects. Furthermore, we analyzed whether KV morphogenesis and ciliogenesis were affected. The results showed that embryos treated with mild cold stress displayed smaller KV than the controls (Figures 3A,B), and the number of cilia decreased greatly, even though no significant change was observed in the length of the cilia (Figures 3C–E). These results demonstrated that mild cold stress led to defects of KV morphogenesis and ciliogenesis in early embryos; these defects may sequentially lead to multiple organ LR patterning defects.

### DFCs clustering defect partially mediates organ LR defects upon mild cold stress

**FIGURE 4:**
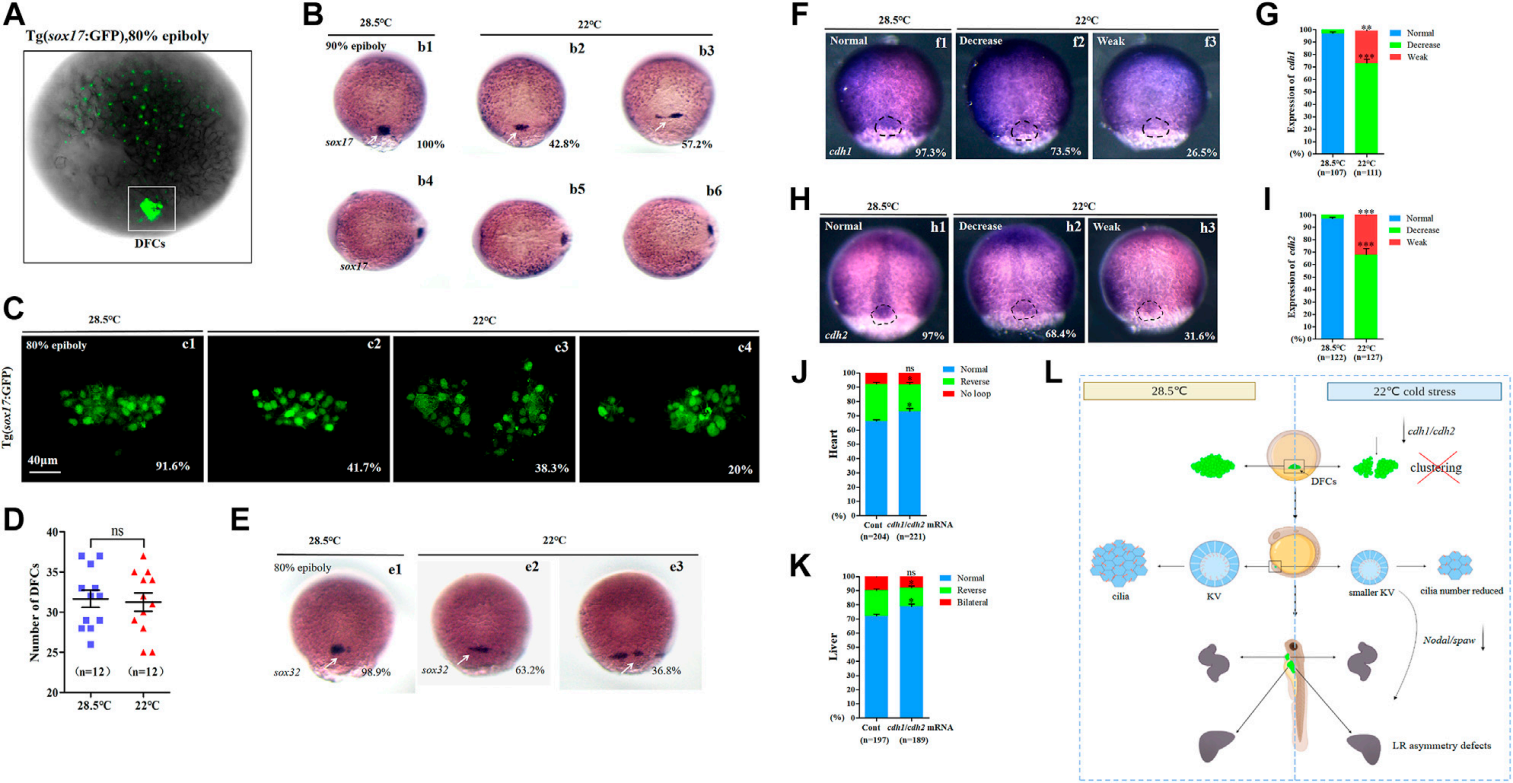
Expression of sox17, sox32, cdh1, and cdh2. **(A)** GFP is expressed in endodermal cells and DFCs at 80% epiboly in Tg (sox17:GFP) transgenic line (DFCs are circled with a white square). **(B)** Expression of sox17 was examined using WISH at 80% epiboly. b1, normal sox17 at 28.5°C (100%, *n* = 67); b2, normal sox17 at 22°C (42.8%, *n* = 74, *p* < 0.002); b3 dispersed expression of sox17 at 22°C (57.2%, *n* = 74, *p* < 0.001). DFCs (white arrow). **(C)** Number DFCs and migration situation of DFCs. C1, DFCs with normal migration at 28.5°C (91.6%, *n* = 12); c2, DFCs with normal migration at 22°C (41.7%, *n* = 12, *p* < 0.003); c3, dispersed DFCs at 22°C (38.3%, *n* = 12, *p* < 0.002); c4: dispersed DFCs at 22°C (20%, *n* = 12, *p* < 0.004). Scale bar, 40 μm. **(D)** Statistical analysis of DFC number. *n* = 12; ns, not significant **(E)** Expression of sox32 at 80% epiboly. e1, normal sox17 at 28.5°C (98.9%, n = 64); e2, normal sox17 at 22°C (63.2%, *n* = 70, *p* < 0.01); e3, dispersed expression of sox17 at 22°C (36.8%, *n* = 70, *p* < 0.03). DFCs (white arrow). **(F)** Expression of cdh1 at 80% epiboly. f1, normal cdh1 at 28.5°C (97.3%, *n* = 107); f2, decreased expression of cdh1 in the DFC field at 22°C (73.5%, *n* = 111, *p* < 0.001); f3, weak expression of cdh1 in the DFC field at 22°C (26.5%, *n* = 111, *p* < 0.01). DFC field (white circle). **(G)** Percentages of normal, decreased, and weak expression of cdh1 in embryos treated at 28.5°C or 22°C. A statistically significant difference (*p* < 0.01) could be seen between the embryos treated at 28.5 vs 22°C. **(H)** Expression of cdh2 in 80% epiboly. h1, normal cdh2 at 28.5°C (97%, *n* = 122); h2, decreased expression of cdh1 in the DFC field at 22°C (68.4%, *n* = 127, *p* < 0.001); h3, weak expression of cdh1 in the DFC field at 22°C (31.6%, *n* = 127, *p* < 0.003). DFC field (white circle). **(I)** Percentages of normal, decreased, and weak expression of cdh2 in embryos treated at 28.5°C or 22°C. A statistically significant difference (*p* < 0.003) could be seen between the embryos treated at 28.5 vs 22°C. **(J)** Percentage of normal looping, reversed looping, and no looping of the heart in embryos treated at 22°C (as control) and embryos treated at 22°C plus overexpression of cdh1/cdh2 mRNA in DFCs. A statistically significant difference (*p* < 0.05) could be seen between control embryos and embryos injected with cdh1/cdh2 mRNA. **(K)** Percentage of normal liver, reversed liver, and liver bifida in embryos treated at 22°C and embryos treated at 22°C plus overexpression of cdh1/cdh2 mRNA in DFCs. A statistically significant difference (*p* < 0.05) could be seen between them. **(L)** Mechanism diagram. Ns, not significant; “*“*p* < 0.05; “**“*p* < 0.03; and “***“*p* < 0.001.

*Sox17* is expressed in endodermal cells and DFCs ((Zhang et al., 2016), Figure 4A). To evaluate whether abnormal KV morphogenesis and ciliogenesis are caused by DFC defects, the expression of *sox17* was examined using *in situ* experiments. The data showed that cells expressing *sox17* were dispersed in 57.2% of embryos upon mild cold stress (Figure 4B, white arrow). We also examined the movement of DFCs and counted DFCs when mild cold stress was applied. It was found that the DFCs migrated in a dispersed manner in 58.3% of embryos (Figure 4C), but the number of DFCs was normal (Figure 4D). To further confirm DFC migration defects, the expression of *sox32*, the other DFC marker gene, was examined. The data showed that 36.8% of embryos displayed serious migration defects upon cold stress (Figure 4E, white arrow). These data indicated that the clustering movement of DFCs, while not the number of DFCs, was disturbed upon mild cold stress.

To evaluate whether defective DFC clustering is related to smaller KV and whether smaller KV correlates with organ LR patterning defects, Tg (*sox17*:GFP) transgenic embryos were treated with mild cold stress, and then they were divided into two groups at 80–90% epiboly: embryos with normal-like DFC clustering and embryos with defective DFC clustering (Supplementary Figure S6A). When the embryos in these two groups developed to the required stages, KV morphogenesis and organ LR patterning were examined. The data showed that the phenotype of KV in embryos with defective DFC clustering became more serious than that in controls, while the phenotype of KV in embryos with normal-like DFC clustering was milder (Supplementary Figures SB,C). Being similar, the ratio of organ LR patterning defects in embryos with defective DFC clustering became higher (Supplementary Figure S6. Dd1, d5–d7; Ff1, f5–f7; E and G), while in embryos with normal-like DFC clustering, it became much lower (Supplementary Figure S6. Dd1–d4; Ff1–f4; E and G). These results implied that DFC clustering defects correlated with KV morphogenesis defects and sequential organ LR patterning defects.

It is well known that normal expression of cell adhesion-relative genes is necessary for the clustering movement of DFCs; disruption of this type of gene leads to defects in the movement of DFCs (Matsui et al., 2011). To find out why DFCs become dispersed upon mild cold stress, we detected the expression of *cdh1* and *cdh2* (Wang et al., 2017). The data showed that the expression of *cdh1* and *cdh2,* especially in DFCs, was downregulated upon mild cold stress (Figures 4F–I). This result implied the possibility that the downregulation of *cdh1/cdh2* in DFCs disturbed DFC clustering during gastrulation. To evaluate this hypothesis, *cdh1* mRNA and *cdh2* mRNA were prepared, then *cdh1* mRNA and *cdh2* mRNA were co-injected at the 256–512 cell stage to cause overexpression in DFCs, and finally, organ LR patterning was analyzed at the required stages. The data showed that the overexpression of *cdh1* and *cdh2* in DFCs partially rescued heart and liver LR patterning defects (Figures 4J,K, Supplementary Figure S7).

In conclusion, all the aforementioned data demonstrated that mild cold stress repressed the expression of *cdh1* and *cdh2*, and this role led to defects in the movement of DFCs and sequential abnormal KV morphogenesis/ciliogenesis and organ LR patterning defects (Figure 4L).

## Discussion

The role of cold stress on organ/tissue development at the zebrafish larval stage has been addressed (Zhang et al., 2018), but the role of cold stress in early development remains unknown. In this study we applied a narrow range of cold stress to early-stage embryos and discovered that these embryos were more sensitive to cold stress (Supplementary Figure S1): treatment with cold stress at 19°C, instead of 6.7°C (Hu et al., 2015), led to embryonic malformation and embryonic death (Supplementary Figure S1 and Table 1). When the temperature rose to 22°C, even though cell-fate determination was not affected, the expression of cell adhesion-related genes was downregulated (Figures 4F–I) and subsequent organ LR patterning was disturbed (Figure 4L), demonstrating a tissue/cell-specific role of cold stress during early embryonic development. Although previous reports have also shown that different organs respond to cold stress with different gene expressions in carp and zebrafish (Gracey et al., 2004; Hu et al., 2015), our research newly identified that the expression of cell adhesion genes was greatly downregulated in DFCs during gastrulation, and this role partially mediated mild cold stress to regulate organ LR patterning.

A range of low-temperature treatments (from 16 to 26°C) led to different degrees of developmental delay (data not shown), but treatment at 24 and 26°C did not result in organ LR patterning defects (Supplementary Table S2); this result ruled out any speculation that developmental delay results in organ LR defects. Furthermore, we found that the cell adhesion genes *cdh1* and *cdh2* were downregulated upon mild cold stress, and overexpression of *cdh1/cdh2* in DFCs partially rescued organ LR patterning defects. This data partially explain how mild cold stress gives rise to organ LR defects. However, earlier studies showed that the expression of a large number of genes was changed by cold stress at the larval or late stage (Long et al., 2012; Long et al., 2013; Hu et al., 2015), which implied the possibility that during gastrulation, the expression of other critical genes was also disturbed upon mild cold stress. Therefore, although our data showed that downregulation of *cdh1/cdh2* in DFCs partially mediated organ LR patterning defects upon cold stress, we could not rule out the role of other types of genes in regulating the movement of DFCs upon mild cold stress, and far more work is needed to elucidate how such stress disrupts the clustering movement of DFCs during gastrulation.

As to the role of cold stress in disease and development, although most previous studies only report its negative role, in fact, a positive role of cold stress has also been observed in some literature (Mollereau, 2015). Recently, the function of endoplasmic reticulum (ER) stress in physiological and pathological conditions has been recognized (Mollereau et al., 2014). ER hormesis is reported to confer neuroprotection by stimulating an autophagic response in mouse and human neuroblastoma models of Parkinson’s disease (Fouillet et al., 2012). Meanwhile, mild hypothermia has been reported to induce an ER stress response and activate the unfolded protein response (UPR) to protect cells from damage of more severe stress (Rzechorzek et al., 2015). Studying the mechanism more closely, mild hypothermia may induce cold shock proteins to confer neuroprotection by increasing structural plasticity at the synapse level (Peretti et al., 2015). Moreover, in addition to the beneficial role of mild cold stress in physiological and pathological conditions, the positive role of mild cold stress was also observed during embryonic development. In zebrafish, *s1pr2* ^AS10^ mutant embryos display cardia bifida, and interestingly, the application of mild cold stress (22.5°C) rescues the cardia bifida phenotype (Lin et al., 2013); the underlying mechanism is that mild cold stress induces ROS to confer upregulation of fibronectin expression, which mitigates symmetric heart progenitor migration in *s1pr2* ^AS10^ mutants.

In summary, even though the role of cold stress in physiology, pathology, and late embryonic development has been broadly addressed (Mollereau, 2015; Reid et al., 2022), the role of cold stress in early embryonic development is largely unknown. Our study newly identified the specific role of mild cold stress in regulating the clustering movement of DFCs and organ LR patterning. Far more work is needed to elucidate the underlying mechanisms of how mild cold stress disturbs the clustering movement of DFCs during gastrulation.

## Data Availability

The original contributions presented in the study are included in the article/Supplementary Material; further inquiries can be directed to the corresponding authors.
