# Supplementary material for: Mild cold stress specifically disturbs clustering movement of DFCs and sequential organ left-right patterning in zebrafish
Source: Front Cell Dev Biol. 2022 Sep 23;10:952844. doi: 10.3389/fcell.2022.952844 (PMC9539758; doi:10.3389/fcell.2022.952844)
Supplement: Supplementary file 1 [file DataSheet1.docx]

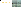
Supplementary Material

**
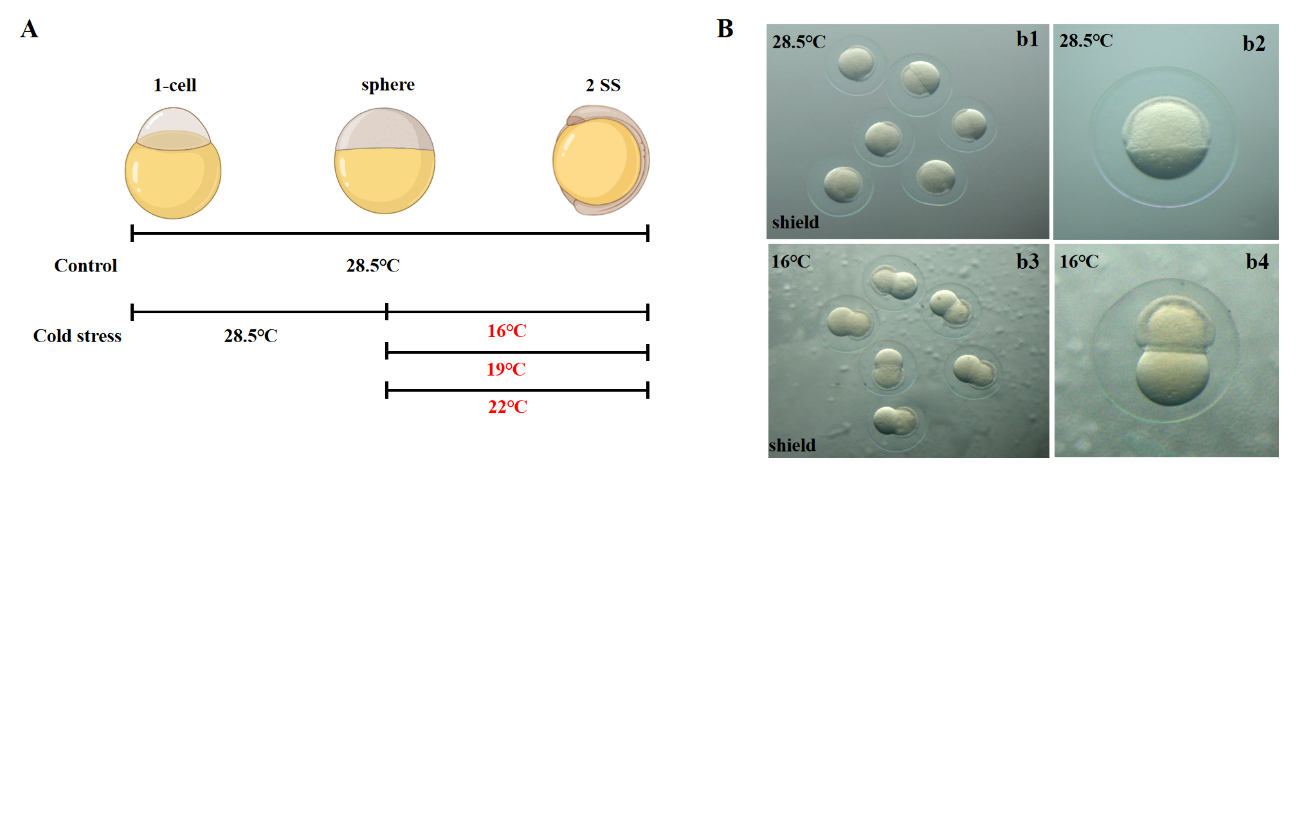
**

**Supplementary Figure 1**. Embryonic deformity and mortality after cold-stress treatment.

(A) The schedule of cold-stress treatment (16 ℃, 19 ℃ 22 ℃). (B) Cold treatment (16 ℃) resulted in deformed embryos during the shield period.

**
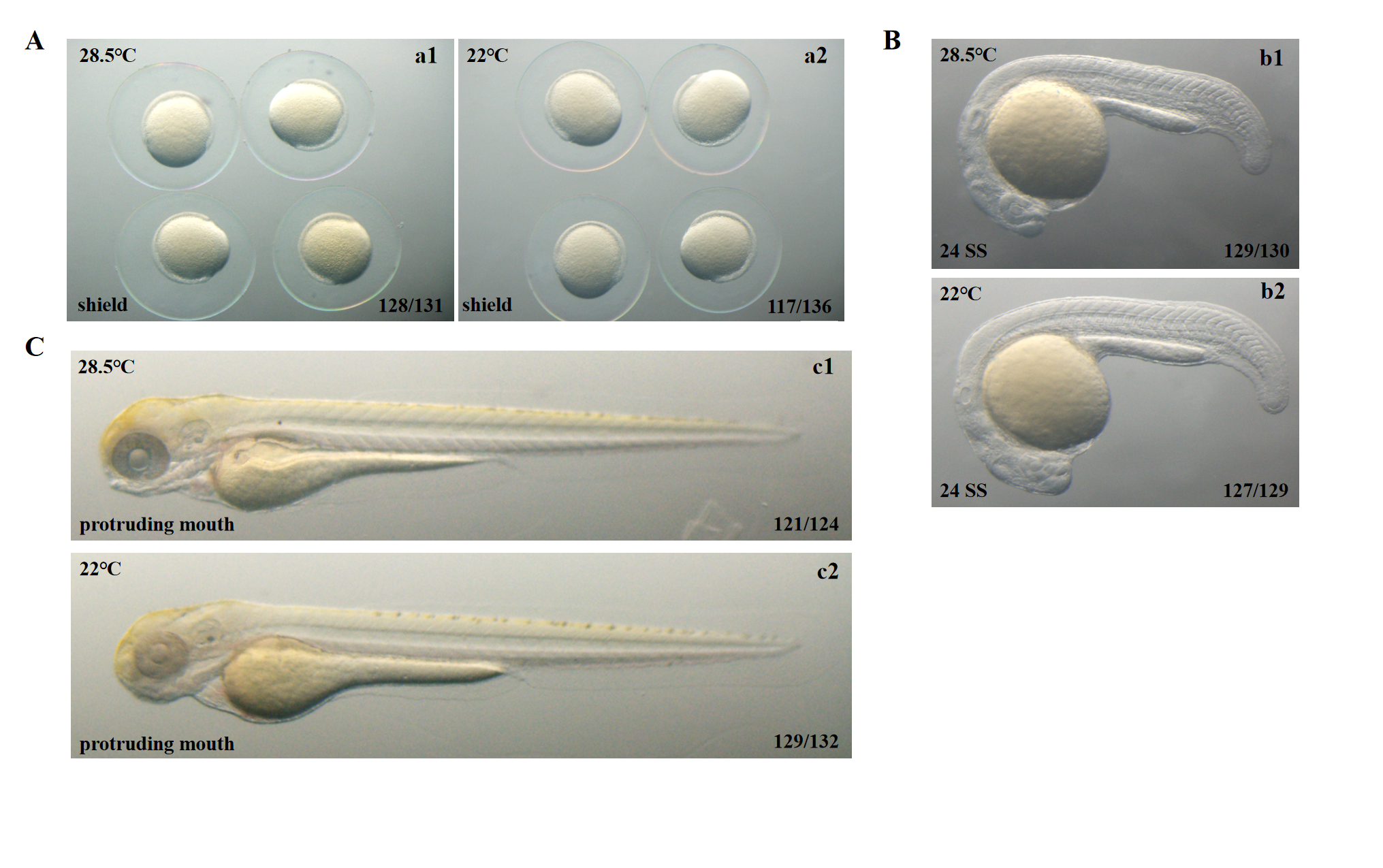
**

**Supplementary Figure 2**. The appearance of embryos treated with mild cold stress.

(A–C) The appearance of embryos treated with cold stress (22 °C) at the shield stage (A), 24 SS (B), protruding mouth stage (C). There was no significant difference between controls and treated embryos.


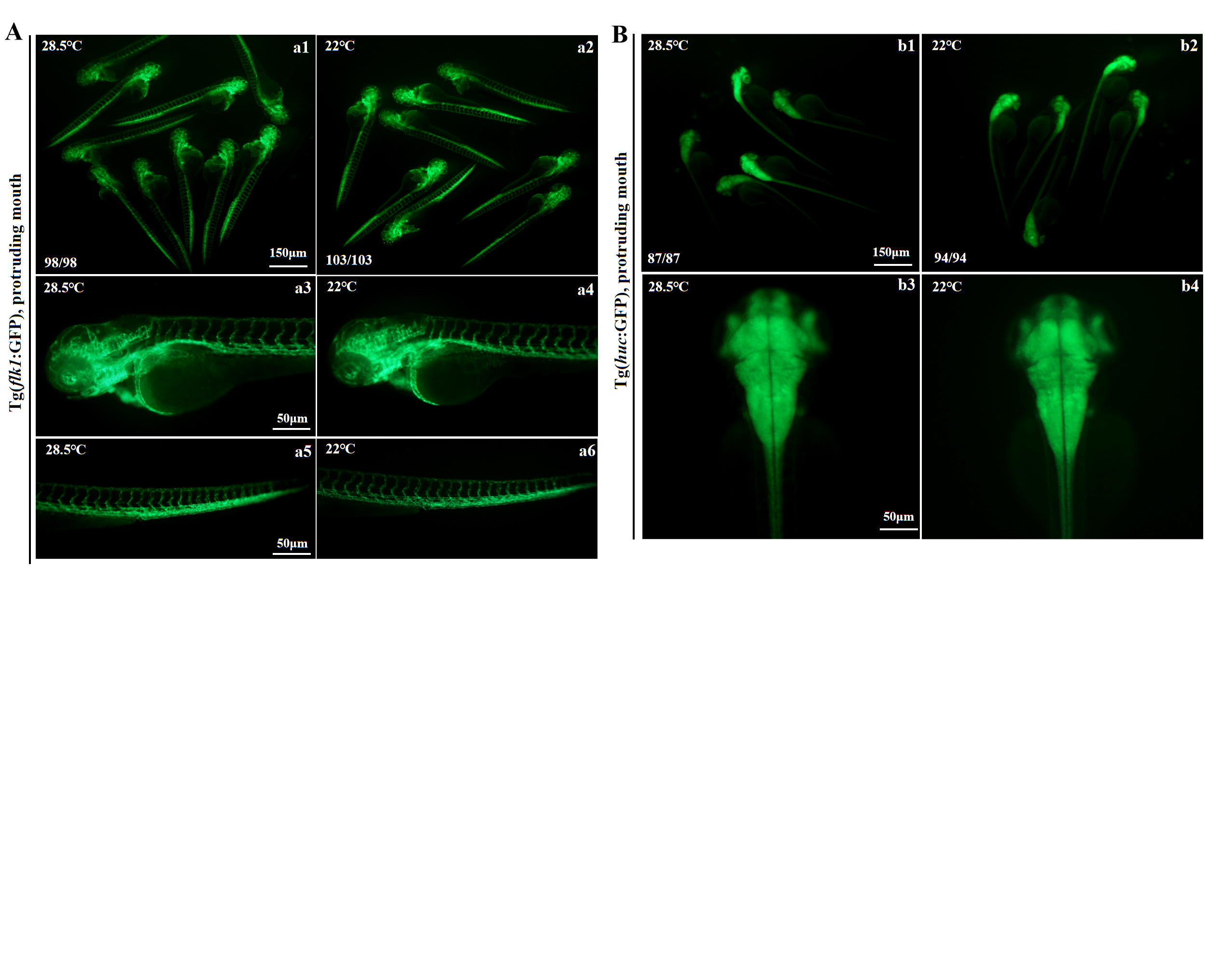


**Supplementary Figure 3**. The development of blood vessels and nervous system in embryos treated with mild cold stress.

(A, B) No obvious defects of blood vessels (A) or neurons (B) were observed in Tg (*flk1 :*GFP) embryos and Tg (*huc* :GFP) embryos treated with cold stress. Scale bar：150 μm (a1, a2, b1, b2), 50 μm (a3–a6, b3, b4).


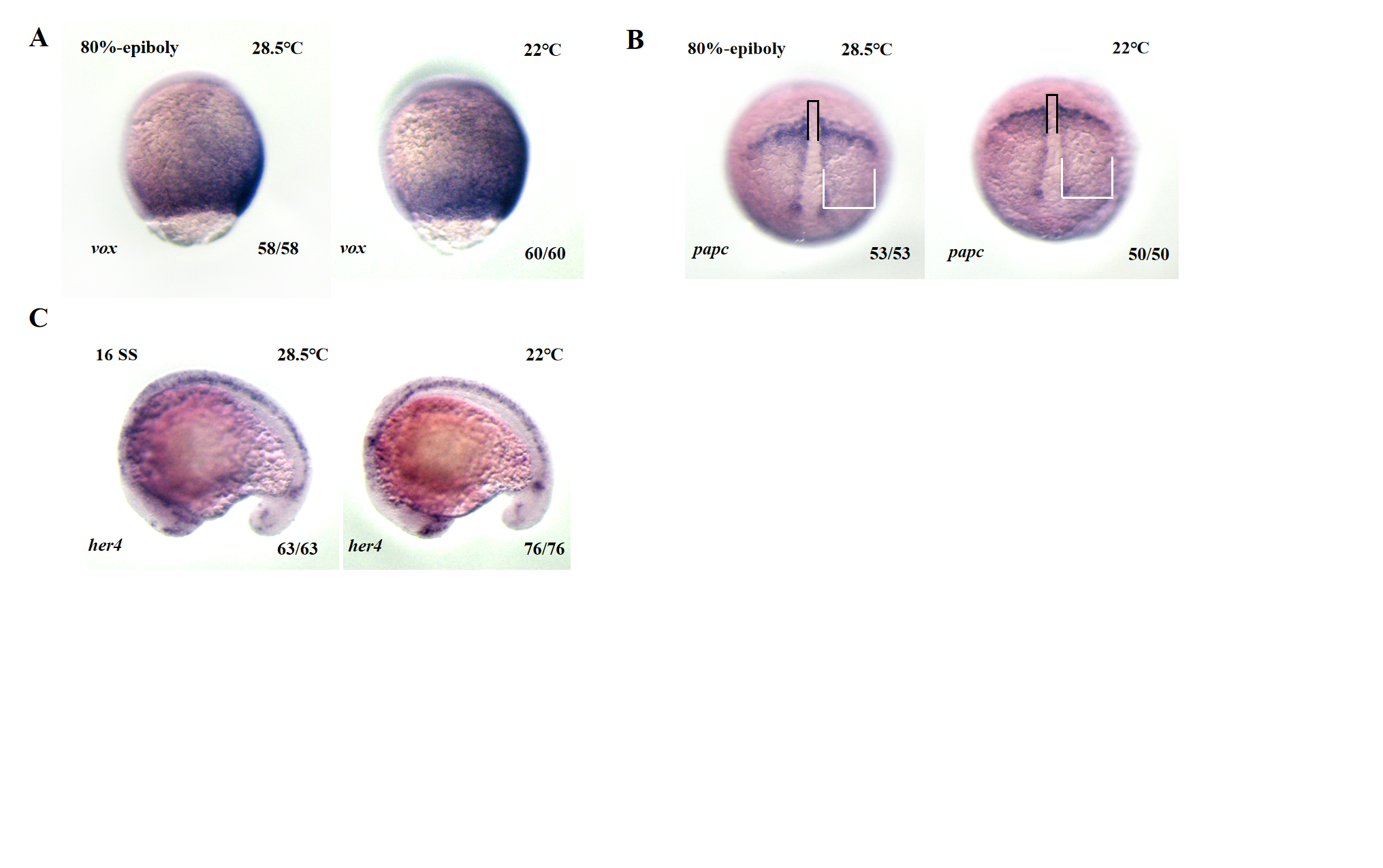


**Supplementary Figure 4**. Mild cold stress did not affect the expression of *vox*, *papc,* and *her4* in early zebrafish embryos.

1. The expression of *vox* in embryos treated with cold stress and control embryos at 80%-epiboly. (B) Expression of *papc* in control and cold-stressed embryos. Notochord (black border), mesoderm (white border). (C) Expression of *her4* in control and cold-stressed embryos.


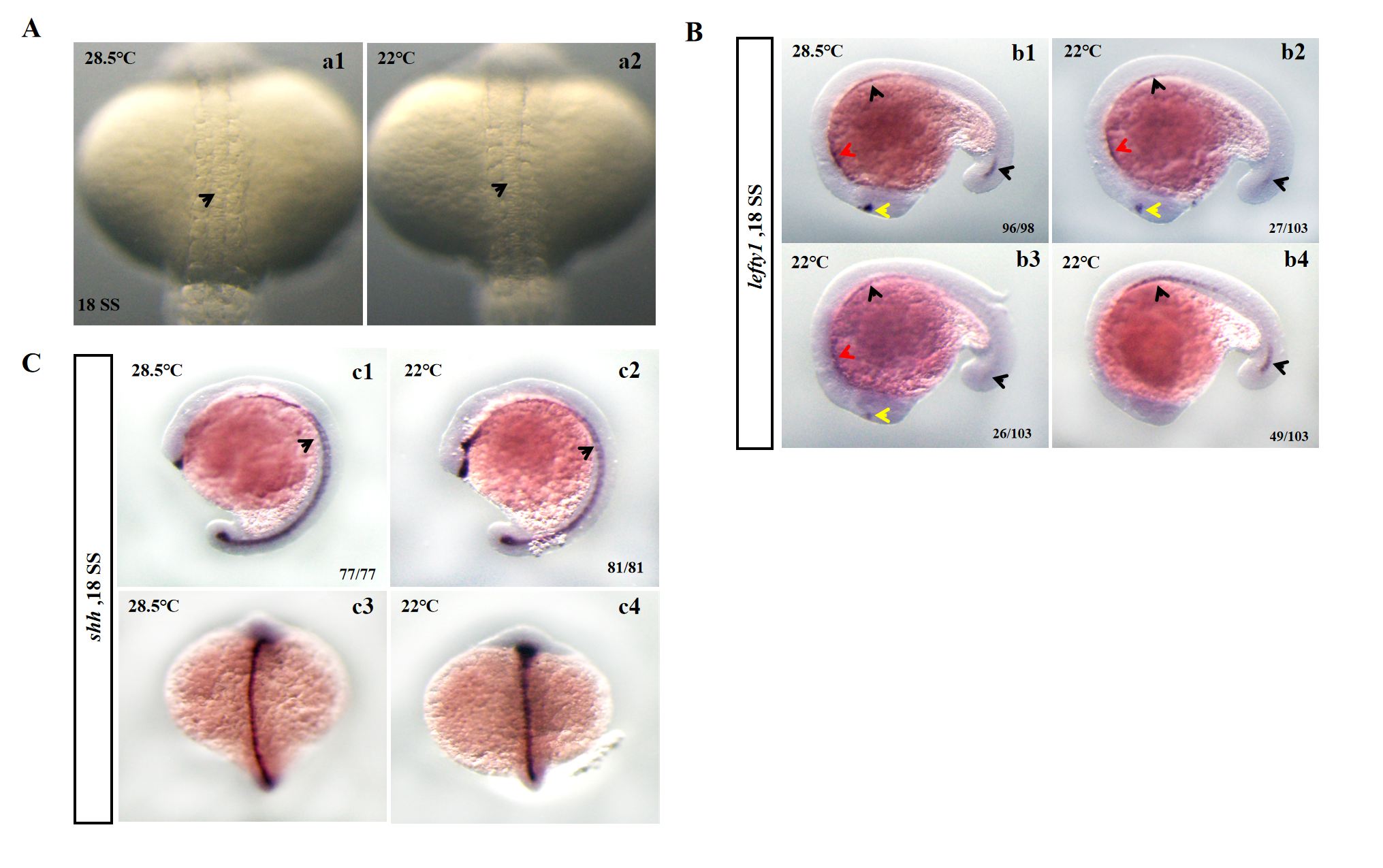


**Supplementary Figure 5.** Mild cold stress does not affect midline formation.

1. The morphology of the dorsal midline (black arrow) was normal in the control embryos and embryos treated with cold stress. (B) *Lefty1* is expressed in the left telencephalon (yellow arrow), left heart field (red arrow), trunk midline, and tail midline (black arrow) at 18 SS. The expression of *lefty1* in the midline was normal in embryos treated with cold stress. (C) The expression of *shh* in the midline remained unchanged in embryos treated with mild cold stress.


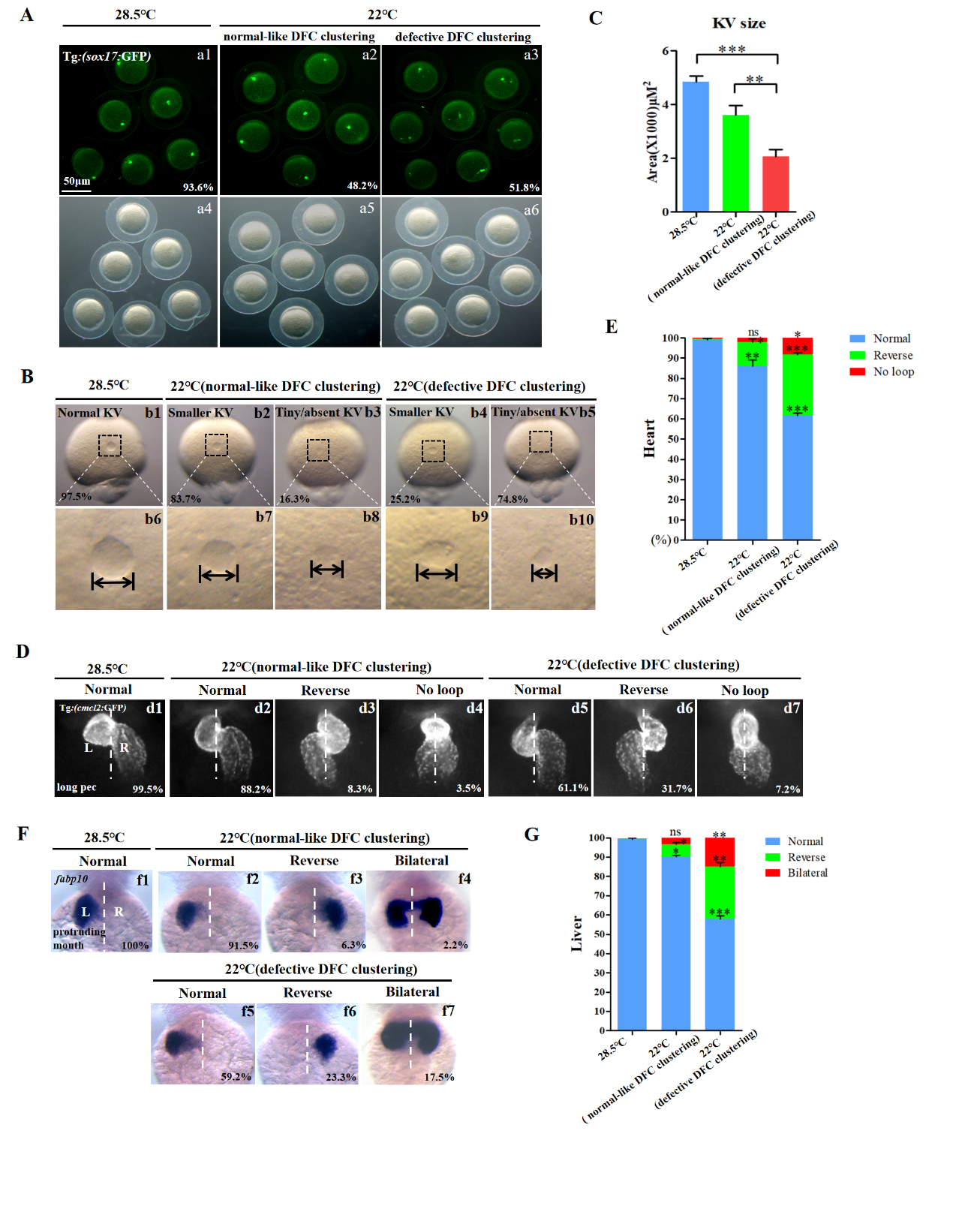


**Supplementary Figure 6.** Defective DFC clustering affects KV morphogenesis and organ LR patterning upon mild cold stress.

(A) The clustering of DFC was observed in Tg (*sox17* :GFP) at the 80%–90% epiboly stage. a1, DFC with normal migration at 28.5 ℃ (93.6%, n=209); a2, normal-like DFC clustering at 22 ℃ (48.2%, n=390, P<0.002); a3, defective DFC clustering at 22 ℃ (51.8%, n=390, P < 0.001); scale bar: 50 μm. (B, C) b1, normal KV at 28.5 ℃ (97.5%, n=198); b2, smaller KV at 22 ℃ (normal-like DFC clustering) (83.7%, n=185, P<0.001); b3, tiny/absent KV at 22 ℃ (normal-like DFC clustering) (16.3%, n=185, P<0.03); b4, smaller KV at 22 ℃ (defective DFC clustering) (25.2%, n=202, P<0.03); b5, tiny/absent KV at 22 ℃ (defective DFC clustering) (74.8%, n=202, P<0.001). A statistically significant (P<0.03) difference could be seen at 28.5 ℃ vs. 22 ℃ (normal-like DFC clustering) and 22 ℃ (defective DFC clustering) embryos. (D, E) Heart morphogenesis in Tg (*cmcl2* :GFP) transgenic line. d1, normal-loop at 28.5 ℃ (99.5%, n=182); d2, normal-loop at 22 ℃ (normal-like DFC clustering) (88.2%, n=175, P<0.03); d3, reversed-loop at 22 ℃ (normal-like DFC clustering) (8.3%, n=175, P<0.05); d4, no loop at 22 ℃ (normal-like DFC clustering) (3.5%, n=175, ns); d5, normal-loop at 22 ℃ (defective DFC clustering) (61.1%, n=180, P<0.001); d6, reversed-loop at 22 ℃ (defective DFC clustering) (31.7%, n=180, P<0.001); d7, no loop at 22 ℃ (defective DFC clustering) (7.2%, n=180, P<0.05). A statistically significant (P<0.05) difference could be seen at 28.5 ℃ vs. 22 ℃ (normal-like DFC clustering) and 22 ℃ (defective DFC clustering) embryos. A statistically significant (P<0.05) difference could be seen at 28.5 ℃ vs. 22 ℃ (normal-like DFC clustering) and 22 ℃ (defective DFC clustering) embryos. (F, G) Liver morphology was examined using *fabp10* probe staining. f1, normal liver in 28.5 ℃ (100%, n=123); f2, normal liver at 22 ℃ (normal-like DFC clustering) (91.5%, n=156, P<0.05); f3, reversed liver at 22 ℃ (normal-like DFC clustering) (6.3%, n=156, P<0.05); f4, liver bifida at 22 ℃ (normal-like DFC clustering) (2.2%, n=156, ns); f5, normal liver at 22 ℃ (defective DFC clustering) (59.2%, n=162, P<0.001); f6, reversed liver at 22 ℃ (defective DFC clustering) (23.3%, n=162, P<0.02); f7, liver bifida at 22 ℃ (defective DFC clustering) (17.5%, n=162, P<0.03). A statistically significant (P < 0.05) difference could be seen at 28.5 ℃ vs. 22 ℃ (normal-like DFC clustering) and 22 ℃ (defective DFC clustering) embryos. Ns, not significant; “*”P＜0.05; “**”P＜0.03; “***”P＜0.001.

**
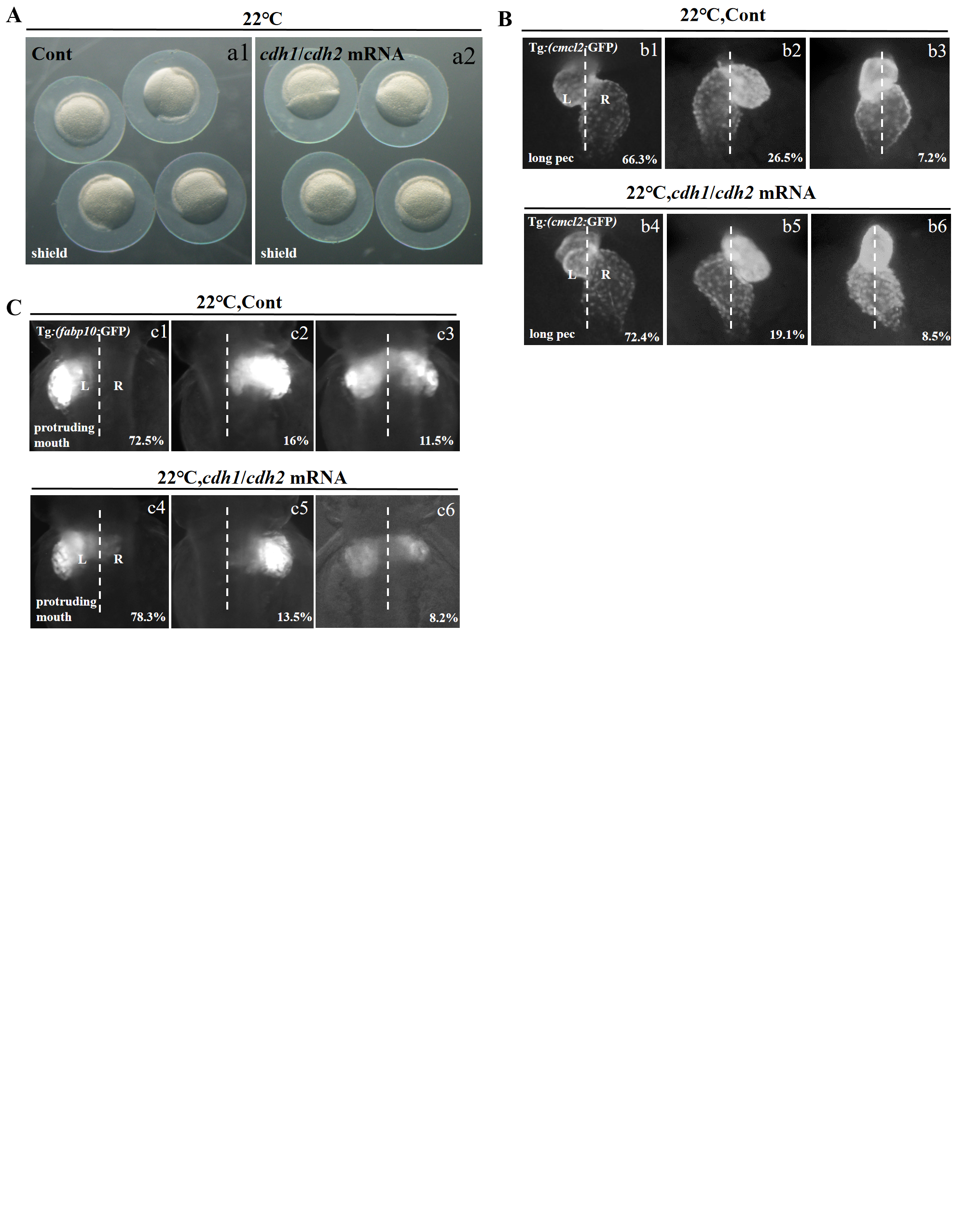
**

**Supplementary Figure 7.** Co-injection of *cdh1*/*cdh2* mRNA partially rescued organ LR patterning defects.

(A) Injection of *cdh1/cdh2* mRNA at 256-512 cell stage did not result in developmental delay or embryonic malformation when compared with controls at the shield stage. (B) Heart morphogenesis in Tg (*cmcl2* :GFP) transgenic embryos. b1, normal-loop at 22 ℃ (66.3%, n=204,); b2, reversed-loop at 22 ℃ (26.5%, n=204); b3, no loop at 22 ℃ (7.2%, n=204); b4, normal-loop at 22 ℃ (72.4%, n=221, P<0.05); b5, reversed-loop at 22 ℃ (19.1%, n=221, P<0.05); b6, no loop at 22 ℃ (8.5%, n=221, ns). (C) Liver morphogenesis in Tg (*fabp10* :GFP) transgenic embryos. c1, normal liver at 22 ℃ (72.5%, n=197,); c2, reversed liver at 22 ℃ (16%, n=197); c3, liver bifida at 22 ℃ (11.5%, n=197); c4, normal liver at 22 ℃ (78.3%, n=189, P<0.05); c5, reversed liver at 22 ℃ (13.5%, n=189, P<0.05); c6, liver bifida at 22 ℃ (8.2%, n=189, ns). Ns, not significant.

**Supplementary Table 1.** Rates of embryonic deformity and mortality upon cold-stress treatment

| **Temperature** | **Deformity rate**  **(shield)** | **Death rate**  **(shield)** | **Deformity rate**  **(2 SS)** | **Death rate**  **(2 SS)** |
| --- | --- | --- | --- | --- |
| 28.5 ℃ | 4% (n=350) | 2% | 3% (n=343) | 13% |
| 22 ℃ | 5% (n=380) | 2% | 4% (n=374) | 15% |
| 19 ℃ | 17% (n=360) | 3% | 9% (n=348) | 36% |
| 16 ℃ | 98% (n=360) | 2% | 0% (n=351) | 100% |

**Supplementary Table 2.** Heart and liver LR patterning defects upon treatment with 24 ℃ or 26 ℃.

| **Temperature** | **Normal**  **(heart)** | **Reverse**  **(heart)** | **No-loop**  **(heart)** | **Normal**  **(liver)** | **Reverse**  **(liver)** | **Bilateral**  **(liver)** |
| --- | --- | --- | --- | --- | --- | --- |
| 24 ℃ | 94.2% (n=208) | 3.4% | 2.4% | 96.5% (n=195) | 3.5% | 0% |
| 26 ℃ | 97.8% (n=231) | 0% | 2.2% | 99% (n=200) | 1% | 0% |
